# The Effects of Time-Restricted Eating on Inflammation and Oxidative Stress in Overweight Older Adults: A Pilot Study

**DOI:** 10.3390/nu17020322

**Published:** 2025-01-17

**Authors:** Armin Ezzati, Javier A. Tamargo, Leah Golberg, Mark D. Haub, Stephen D. Anton

**Affiliations:** 1 Department of Physiology and Aging, College of Medicine, University of Florida, Gainesville, FL 32611, USA; j.tamargo@ufl.edu (J.A.T.);; 2 Department of Food Nutrition Dietetics and Health, Kansas State University, Manhattan, KS 66506, USA; haub@ksu.edu; 3 Department of Clinical and Health Psychology, University of Florida, Gainesville, FL 32611, USA

**Keywords:** inflammation, intermittent fasting, older adults, oxidative stress, time-restricted eating

## Abstract

Background/Objectives: Time-restricted eating (TRE) has been associated with beneficial effects for inflammation and oxidative stress; however, the effects of TRE on inflammation and oxidative stress in the aging population have not been explored. Methods: This secondary analysis tested the effects of TRE on pro-inflammatory (hs-CRP [high-sensitivity C-reactive protein], IL-1β [interleukin 1 beta], IL-6 [interleukin 6], TNF-α [tumor necrosis factor alpha]) and oxidative stress (8-isoprostane) biomarkers in ten overweight older adults (mean age = 77.1 ± 6.1 years; six women and four men), who followed a TRE protocol of 16 h of fasting per day and consumed food ad libitum during an 8 h window for 4 weeks. Results: TNF-α levels decreased from 43.2 (11.2) pg/mL to 39.7 (10.0) pg/mL with a Cohen’s d effect size of 0.33, and IL-1β levels decreased from 1.4 (0.8) pg/mL to 1.3 (0.6) pg/mL with a Cohen’s d effect size of 0.23, suggesting potential anti-inflammatory benefits. IL-6 and hs-CRP levels showed no substantial changes (Cohen’s d ≤ 0.03). The oxidative stress marker 8-isoprostane levels decreased slightly with a Cohen’s d effect size of 0.07. Conclusions: The findings of this pilot study provide initial insights into the potential effects of TRE on inflammatory and oxidative stress markers in older adults. Given the small sample size and short-term intervention, well-powered studies of longer duration are needed to better understand the effects of TRE on inflammation and oxidative stress in aging populations.

## 1. Introduction

Aging is accompanied by gradual biological and cellular changes that result in functional decline and significant increases in the risk of age-related diseases [1]. Among these changes is chronic inflammation, which is considered a hallmark of aging [2]. Similarly, oxidative stress, a key driver of age-related diseases, increases with aging [3]. Calorie restriction (CR) has been shown to lower inflammation and oxidative stress [4,5]. However, challenges in adherence and daily caloric intake monitoring necessitate alternative approaches [6].

Intermittent fasting (IF) is an umbrella term that describes an eating pattern in which an individual fasts for an extended time-period, typically longer than 12 h, on a repeated basis [7]. Of the various types of IF approaches, time-restricted eating, the pattern of extending the daily fasting period and thereby reducing the daily eating period, has gained popularity over the last few years [8]. Time-restricted eating (TRE) has shown effectiveness as a viable dietary approach for improving body composition [9,10], metabolic parameters [10,11], inflammatory markers [12,13,14,15], and oxidative stress markers [15,16,17]. Some studies reported a reduction of 200–500 kcal daily with TRE without the need for calorie counting [18,19,20]. While the effects of TRE on markers of inflammation and oxidative stress in young to middle-aged populations have been explored by previous studies [13,16,17], no study to date has specifically examined the effects of TRE with ad libitum intake on inflammatory markers and oxidative stress in older adults.

Therefore, the primary aim of this pilot study was to evaluate the effects of a short-term TRE intervention on changes in markers of inflammation, including tumor necrosis factor alpha (TNF-α), interleukin 1 beta (IL-1beta), interleukin 6 (IL-6), high-sensitivity C-reactive protein (hs-CRP), and markers of oxidative stress, 8-isoprostane, in overweight, older adults. We hypothesized that short-term TRE would reduce both markers of inflammation and oxidative stress in overweight, older adults.

## 2. Materials and Methods

### 2.1. Study Design

This pilot study aimed to assess the effects of TRE on inflammatory markers (IL-1β, IL-6, TNF-α) and oxidative stress (8-isoisoprostane) in 10 overweight, older adults from a single-arm intervention, as previously described [21]. The primary goal of the study was to assess the feasibility and safety of TRE in overweight, sedentary older adults over a four-week period. Participants were included if they were aged ≥ 65 years, self-reported difficulty walking ¼ mile or climbing a flight of stairs, were sedentary (<30 min of structured exercise per week), had a walking speed <1 m/s on the 4 m walk test, and had a body mass index between 25 and 40 kg/m^2^ (inclusive). Potential participants were excluded if the screening process—including a phone interview, medical history review, or clinical examination—identified any of the following: fasting for more than 12 h daily, active or planned weight loss efforts, weight loss exceeding 5 lbs in the past month, a resting heart rate above 120 beats per minute, systolic blood pressure greater than 180 mmHg, diastolic blood pressure exceeding 100 mmHg, recent unstable angina, heart attack or stroke (within the past three months), reliance on supplemental oxygen for chronic pulmonary conditions or heart failure, or chronic conditions such as rheumatoid arthritis, Parkinson’s disease, dialysis dependence, or active cancer treatment within the past year. Additionally, participants were excluded if they had insulin-dependent diabetes mellitus, used medications incompatible with a 16 h fasting regimen, or had any medical condition deemed by the investigator to impair trial participation or pose a personal health risk. Any changes in health conditions, including infections or acute conditions that could potentially influence the inflammatory markers measured, were monitored through weekly phone contacts. No infections or acute illnesses were reported by participants during the study period.

### 2.2. Intervention

Study participants were asked to fast for a target of 16 h per day for a period of 4 weeks, and they were allowed to eat ad libitum during the daily 8 h eating window. The first week involved a ramp-up to a full 16 h fasting period (days 1–3: fast for 12–14 h per day; days 4–6: fast for 14–16 h per day; days 7–28: fast for 16 h per day). The study imposed an ad libitum dietary intake, allowing participants to freely determine their meal composition and caloric intake within the TRE window. This approach aimed to facilitate adherence and reflect real-world conditions. Participants were allowed to consume calorie-free beverages, tea, black coffee, and sugar-free gum, and were encouraged to drink plenty of water throughout the intervention period. Participants were asked to record the time of first and final food/drink consumption each day. All testing sessions were performed at the Institute on Aging, University of Florida. The fasting times were self-selected by participants and were allowed to vary daily.

### 2.3. Measures

Fasting blood samples were collected at baseline and the 4-week follow-up visits for inflammatory and oxidative stress biomarkers. Inflammatory cytokines, including IL-1β, IL-6, and TNF-α, were assessed using the Magnetic Luminex Assay Human Premixed Multi-Analyte Kit (R&D Systems, Minneapolis, MN, USA). Human Quantikine ELISA (R&D Systems) was performed to measure hs-CRP levels. The oxidative stress marker 8-isoprostane was measured by 8-iso-Prostaglandin (CELL BIOLABS, San Diego, CA, USA).

### 2.4. Statistical Analysis

Data were checked for missing values and outliers. Any missing data, which occurred due to values falling below the detection limit, were excluded from the analysis. Means and standard deviations (SD) were calculated for each marker per time-point. Primary analyses were performed using SAS software, version 9.4 (SAS Institute Inc., Cary, NC, USA). Statistical analysis included paired sample *t*-tests to compare baseline and week four values for each marker. Changes from baseline to week four were calculated for each outcome and expressed as mean difference and percentage change. Effect sizes were calculated using Cohen’s d to measure the magnitude of the changes. Given the small sample size and focus on feasibility in this pilot study, hypothesis testing may not be adequate and can be misleading. As such, we report *p*-values but warrant caution in their interpretation.

## 3. Results

### Changes in Inflammatory and Oxidative Stress Markers

**Figure 1:**
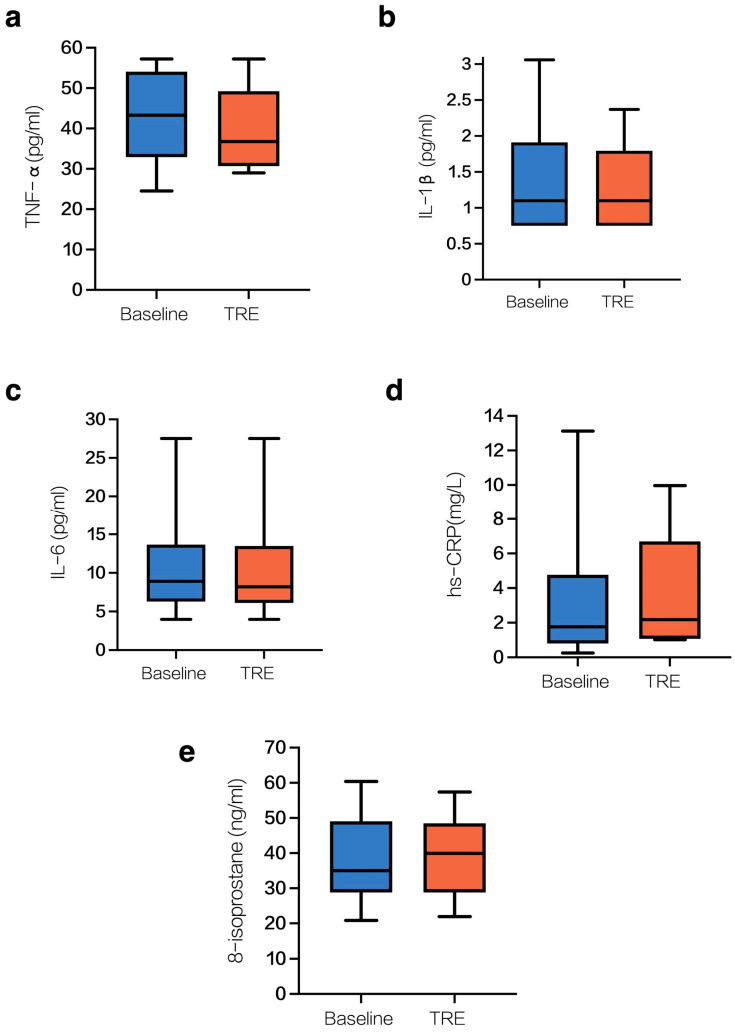
Box-and-whisker plots showing the distribution of data for inflammatory markers and oxidative stress markers at baseline and after 4 weeks of time-restricted eating (TRE). The plots are labeled as follows: (**a**) TNF-α, tumor necrosis factor alpha, (**b**) IL-1β (Interleukin-1 beta), (**c**) IL-6 (Interleukin-6), (**d**) hs-CRP (high-sensitivity C-reactive protein), and (**e**) 8-Isoprostane.

Figure 1 shows the changes in inflammatory and oxidative stress markers from baseline to the end of 4 weeks of time-restricted eating. There were modest reductions in IL-1 b and TNF-α, with effect sizes ranging between 0.23 and 0.33. We did not observe similar changes in other markers of inflammation of oxidative stress. Table 1 reports changes in inflammatory and oxidative stress markers from baseline to the end of 4 weeks. No significant changes were observed in any of the markers at the 4-week time-point (all *p*-values > 0.05).

## 4. Discussion

To our knowledge, this is the first study investigating the effects of TRE with ad libitum intake on markers of inflammation and oxidative stress specifically in sedentary, overweight older adults. The main finding of this study was that TRE produced modest reductions in two pro-inflammatory makers, namely TNF-alpha and IL-1B (based on effect sizes), but did not induce changes in other markers of inflammation, hs-CRP and IL-6, or oxidative stress (8-isoprostane). Given the short-term nature of this study and small sample size, it is possible that TRE may significantly reduce markers of inflammation in adequately powered and longer-duration studies among older populations.

Previous studies have examined the effects of TRE on pro-inflammatory markers in adults with or without obesity [12,13,14,16,17]. In a short-term randomized controlled trial (RCT) [12], TNF-α significantly decreased only with early TRE (6 a.m.–3 p.m.) but not with mid-day TRE (11 a.m.–8 p.m.) in young, healthy individuals without obesity. In another short trial, Sutton et al. tested early TRE (last meal before 3:00 p.m.) in men with pre-diabetes over five weeks and observed no significant changes in IL-6 and hs-CRP levels [17]. Additionally, an eight-week trial of late TRE with longer fasting durations (18 and 20 h) did not alter TNF-α or IL-6 compared to a control in adults with obesity [16].

In contrast to short-term studies, a longer-term study of one-year duration reported a significant reduction in TNF-α, IL-1β, and IL-6 with late TRE (three meals: 1 p.m., 4 p.m., and 8 p.m.) compared to a control group in healthy resistance-trained males [13]. These findings align with the modest improvements in TNF-α and IL-1β observed in our study, which imposed self-selected eating windows with TRE. Specifically, our four-week TRE intervention showed small effect sizes for reductions in TNF-α and IL-1β, suggesting that even a shorter duration of TRE may initiate positive shifts in inflammatory markers, particularly in aging populations. While these effects were modest and non-significant, they highlight the potential of TRE to impact systemic inflammation over time. The small effect sizes observed may illustrate early, subtle changes in inflammatory pathways that could potentially induce more pronounced effects with longer interventions or higher adherence to TRE protocols. Furthermore, these findings highlight the possibility that older adults, who often exhibit heightened levels of chronic, low-grade inflammation (inflammaging), may respond differently to TRE compared to younger populations.

Our null findings with IL-6 and hs-CRP are also consistent with some prior short-term studies. Martens et al. [22] examined eucaloric TRE (16:8), without weight loss, in 22 healthy non-obese middle-aged and older adults in a RCT. After 6 weeks, there were no significant differences between TRE and normal diet control for IL-6 and CRP levels [22]. Similarly, others have found no significant changes in IL-6 and CRP/hs-CRP levels with short-term TRE interventions (5 to 8 weeks) [11,16,17]. In contrast, early 16:8 TRE (eating window between 8 a.m and 4 p.m.) resulted in significant reductions in hs-CRP levels (42%) in overweight women with polycystic ovary syndrome over 5 weeks [14]. In addition, the biological variability of the inflammatory markers assessed in this study (hs-CRP and interleukins) may have influenced our findings [23]. Inflammatory marker levels can exhibit significant fluctuations due to various biological factors, including genetic variability [24,25] and transient physiological conditions (e.g., acute stress) [26].

To our knowledge, the present study is the first to examine a marker of oxidative stress following 16:8 TRE. Oxidative stress marker 8-isoprostane slightly decreased after 4 weeks of TRE. In contrast, longer daily fasting durations of 18–20 h have been shown to lower 8-isoprostane significantly in pre-diabetic men or adults with obesity after 5 and 8 weeks, respectively [16,17]. Notably, 18:6 early TRE significantly reduced the oxidative stress marker 8-isoprostane, even in the absence of weight loss [17]. Additionally, late TRE with longer fasting durations (18 and 20 h) reduced oxidative stress 8-isoprostane in adults with obesity following 8 weeks [17]. In the secondary analysis of the study [27], however, reductions in oxidative stress were more closely related to the degree of weight and fat loss (>3.5% weight loss). Our study [21] observed lower weight loss (~3% of initial body weight), implemented 16 h TRE, and had a shorter time frame, which may partly explain the unchanged levels of oxidative stress.

### 4.1. Future Directions

The current pilot study utilized TRE with self-selected eating windows, with the average eating time occurring 61 min later at week 4 compared to week 1 (mean start time: 9:32 a.m. in week 1 vs. 10:33 a.m. in week 4) [28]. In contrast, a 5-week early TRE intervention (6 a.m. to 3 p.m.) improved pro-inflammatory cytokines (TNF-α and IL-8) in young adults without obesity, while midday TRE (11 a.m. to 8 p.m.) did not show improvements in these markers [12]. Future research should investigate the effects of early TRE and different meal timings within TRE protocols on the aging population.

Diet is closely associated with inflammation and oxidative stress [29]. Systematic reviews and meta-analyses suggest that some dietary patterns, such as the Mediterranean diet, may reduce levels of pro-inflammatory markers and thereby contribute to healthier aging in older adults [30,31,32]. In contrast, diets high in ultra-processed foods, added sugars, and unhealthy fats are linked to elevated levels of inflammatory markers [33,34,35]. The dietary patterns of participants in this study may have influenced the changes in inflammatory and oxidative stress markers; however, we did not control for diet quality or assess specific dietary patterns. Thus, it remains uncertain to what extent variability in inflammatory responses was driven by individual dietary habits.

Future research would benefit from examining the combined effects of TRE with different dietary patterns on markers of inflammation and oxidative stress in older adults. Studies targeting the aging population should also consider employing a eucaloric isocaloric cross-over design, where all participants consume standardized meals with similar amounts of calories, micronutrients, and macronutrients, engaging in both TRE and a control condition with eating windows spread out over the entire day. This approach would allow for a direct within-participant comparison, minimizing inter-individual variability, and would provide clearer insights into the independent effects of TRE on inflammatory and oxidative stress markers. Additionally, longer and more robust trials, incorporating comprehensive dietary assessments, advanced monitoring tools, and consideration of other lifestyle factors, are essential to more clearly elucidate the role of TRE in mitigating age-related inflammation and oxidative stress. In addition to meal timing, fasting duration may also affect changes in markers of inflammation and oxidative stress. However, the current study examined a 16 h TRE, which may not have been sufficient to fully capture the effects within this study’s shorter timeframe. Future well-powered studies with longer fasting durations could provide further clarity on the optimal timing and duration to maximize the health benefits of TRE in older adults. Additionally, the effects of TRE interventions on other hallmarks of aging, including autophagy, epigenetic alterations, and cellular senescence, should be investigated in future trials.

### 4.2. Strengths and Limitations

This was the first trial to explore the effects of TRE with ad libitum intake on inflammation and oxidative stress in older adults. The strength of this study is the reporting of effect sizes, which provide a nuanced understanding of the potential impact of TRE beyond statistical significance. The inclusion of effect size analysis is particularly valuable in pilot studies with small sample sizes, as it helps in accessing the magnitude of observed changes for the design of future, larger trials. The study is also strengthened by high levels of adherence, which may be partly due to self-selected eating windows. Participants reported an adherence rate of 84% through self-reported diaries [28].

The study has several limitations, and the pilot nature of the study, its focus on feasibility, the small sample size, and the short duration warrant careful interpretation of the results. Although common in dietary interventions, self-reporting can introduce potential limitations due to recall bias and social desirability bias. Additionally, while self-selected eating windows may enhance adherence to TRE, they may also hinder the ability to assess the effects of meal timing (early, midday, late) on health outcomes. Furthermore, while the ad libitum intake approach was intentionally selected to enhance adherence and practicality, reflecting real-world applications of TRE, the lack of dietary intake assessment and calorie control limits the generalizability of the study findings. Variability in diet quality and caloric intake could have mediated the effects of TRE on inflammatory and oxidative stress markers. Notably, given the exploratory nature of this study, we did not restrict our inclusion criteria due to smoking and excessive alcohol consumption; however, such factors ought to be considered exclusionary in future studies. These limitations call for longer, more robust trials with comprehensive dietary assessments and advanced monitoring tools to better elucidate the impact of TRE in mitigating age-related inflammation and oxidative stress.

## 5. Conclusions

In conclusion, this pilot study provides initial insights into the potential effects of TRE on inflammation and oxidative stress in overweight older adults. While the small effect sizes observed suggest early, subtle changes in inflammatory pathways, the findings underscore the feasibility of TRE in this population, supported by high adherence rates. However, given the small sample size and short duration of the study, this finding should be interpreted with caution. Future well-powered studies with longer durations are needed to elucidate the effect of TRE on inflammation and oxidative stress in older populations.

## Figures and Tables

**Table 1:** Changes in inflammatory and oxidative stress markers from baseline to the end of 4 weeks of time-restricted eating (TRE). Values are presented as mean (standard deviation), with Δ Mean representing the difference from baseline. Percentage changes reflect the relative change from baseline to TRE. Cohen’s d (effect size) indicates the magnitude of change, and *p*-value denotes statistical significance.

Study Measures	Baseline M (SD)	TRE M (SD)	Δ Mean (SD)	%Change	Cohen’s d	*p*-Value
TNF-alpha (pg/mL)	43.2 (11.2)	39.7 (10.0)	−3.5 (5.7)	−8%	0.33	0.101
IL-1β (pg/mL)	1.4 (0.8)	1.3 (0.6)	−0.1 (0.7)	−11%	0.23	0.499
IL-6 (pg/mL)	10.8 (7.2)	10.8 (7.2)	−0.001 (2.4)	0%	0.03	0.998
hs-CRP (mg/L)	3.3 (4.1)	3.6 (3.4)	0.3 (1.8)	10%	0.01	0.596
8-isoprostane (ng/mL)	41.3 (13.8)	38.7 (11.6)	−0.4 (12.2)	−1%	0.07	0.919

## Data Availability

The data presented in this manuscript may be available upon request, subject to the submission of an application and approval by the corresponding author. The data are not publicly available due to ethical reasons.
